# Clinical application of transoral and submental thyroidectomy (TOaST): a series of 54 human cases

**DOI:** 10.1007/s00423-022-02663-w

**Published:** 2022-08-29

**Authors:** Hang Chen, Lijuan Deng, Keyi Xu, Zhixian Gong, Xiaoping Zhu

**Affiliations:** 1 Department of Day Ward, The First Affiliated Hospital of Nanchang University, Nanchang, 330006 Jiangxi China; 2 The Nephrology Department, The First Affiliated Hospital of Nanchang University, Nanchang, 330006 Jiangxi China

**Keywords:** Endoscopic thyroidectomy, Papillary thyroid carcinoma, TOaST, Transoral endoscopic thyroidectomy

## Abstract

**Objective:**

A new endoscopic thyroidectomy approach**—**transoral and submental endoscopic thyroidectomy (TOaST)—was applied in clinical practice and considered an improved approach for endoscopic thyroid surgery via the oral approach. This paper discusses the feasibility and effectiveness of this surgical method.

**Methods:**

A retrospective analysis was performed on the clinical data of 54 patients who had undergone TOaST in the thyroid disease center of the First Affiliated Hospital of Nanchang University between December 2020 and December 2021. The surgical data and techniques, complications, and cosmetic outcomes of these patients were studied.

**Results:**

Among the total 54 patients, 23 underwent unilateral subtotal thyroidectomy, 3 patients underwent bilateral subtotal thyroidectomy, 27 with unilateral thyroid cancer underwent affected thyroid + isthmus + central lymph node resection, and only 1 patient underwent total thyroidectomy. The mean operative time was 88.06 ± 12.03 min (range: 65–135 min), the mean intraoperative blood loss was 8.61 ± 4.60 ml (range: 5–20 ml), the mean postoperative drainage volume was 49.96 ± 9.88 ml (range: 30–60 ml), the mean drainage time was 36.61 ± 2.65 h (range: 32–50 h), and the mean length of hospital stay was 46.63 ± 3.28 h (range 45–70 h). One patient experienced transient recurrent laryngeal nerve injury, and another patient experienced transient parathyroid dysfunction; there was no superior laryngeal nerve injury and other complications, such as postoperative subcutaneous hematoma, hypercapnia, mental nerve injury, tracheoesophageal injury, infection, or lymphatic leakage.

**Conclusion:**

TOaST cannot only achieve a good therapeutic effect but also avoid mental nerve injury, reduce the discomfort of the patient’s jaw, obtain a good cosmetic effect, and facilitate the operation of the operator. It is an endoscopic thyroidectomy technique with a certain clinical value.

**Supplementary Information:**

The online version contains supplementary material available at 10.1007/s00423-022-02663-w.

## Introduction

Transoral endoscopic thyroidectomy has been developed and carried out by many thyroid surgeons because of its advantages, such as short path, no surgical scar on the body surface, treatment of bilateral thyroid glands, adherence to the principle of natural orifice transluminal endoscopic surgery (NOTES), and convenience for thorough dissection of lymph nodes in the central region [1, 2]. After more than 10 years of development, transoral endoscopic thyroidectomy has been improved from the initial oral floor approach to the oral vestibular approach, which effectively solves oral discomfort and postoperative difficulty while eating. It also makes transoral endoscopic thyroidectomy one of the best surgical methods with a comprehensive cosmetic effect and excision effect [3, 4]. However, as the turning point of the mandible is the lever support point of the central trocar, the surgeon needs to make a larger incision when establishing the central trocar channel, and the mandibular muscles can be over-stretched or torn during the operation, which will undoubtedly aggravate the postoperative mandibular discomfort of patients. Moreover, being the narrowest part of the passage, the turning point of the mandible makes it more difficult to remove the specimen [5]. Accordingly, Chen et al. set the central trocar incision at the mandibular base, thus avoiding the turn of the mandible. Thus, transoral and submental endoscopic thyroidectomy (TOaST) was designed [6]. Our research group reached a consensus with Chen et al. on this issue, and we herein report our study on TOaST.

## Materials and methods

A retrospective analysis was performed on the clinical data of 54 patients who had undergone TOaST in the thyroid disease center of the First Affiliated Hospital of Nanchang University between December 2020 and December 2021. All patients were informed about the surgical procedure and signed consent forms before surgery. All of the surgeries were performed by the same physician experienced in endoscopic thyroid surgery. The ethics committee of the First Affiliated Hospital of Nanchang University approved this study.

The inclusion criteria were as follows: (1) the patient had cosmetic needs and requested endoscopic thyroidectomy; (2) the diameter of benign nodules was less than 4 cm, and that of cystic nodules was less than 6 cm; (3) in the case of differentiated thyroid carcinoma (DTC), a nodule less than 2 cm in diameter, without local invasion and obvious lymph node metastasis.

Separately, the study exclusion criteria were as follows: (1) with extensive thyroid inflammation; (2) general anesthesia was not tolerated; (3) history of neck surgery or radiation; (4) preoperative evaluation showed that the tumor invaded the recurrent laryngeal nerve (RLN) or was located where the nerve entered the larynx; (5) patients with hyperthyroidism without any medication control.

### Surgical technique

**Fig. 1:**
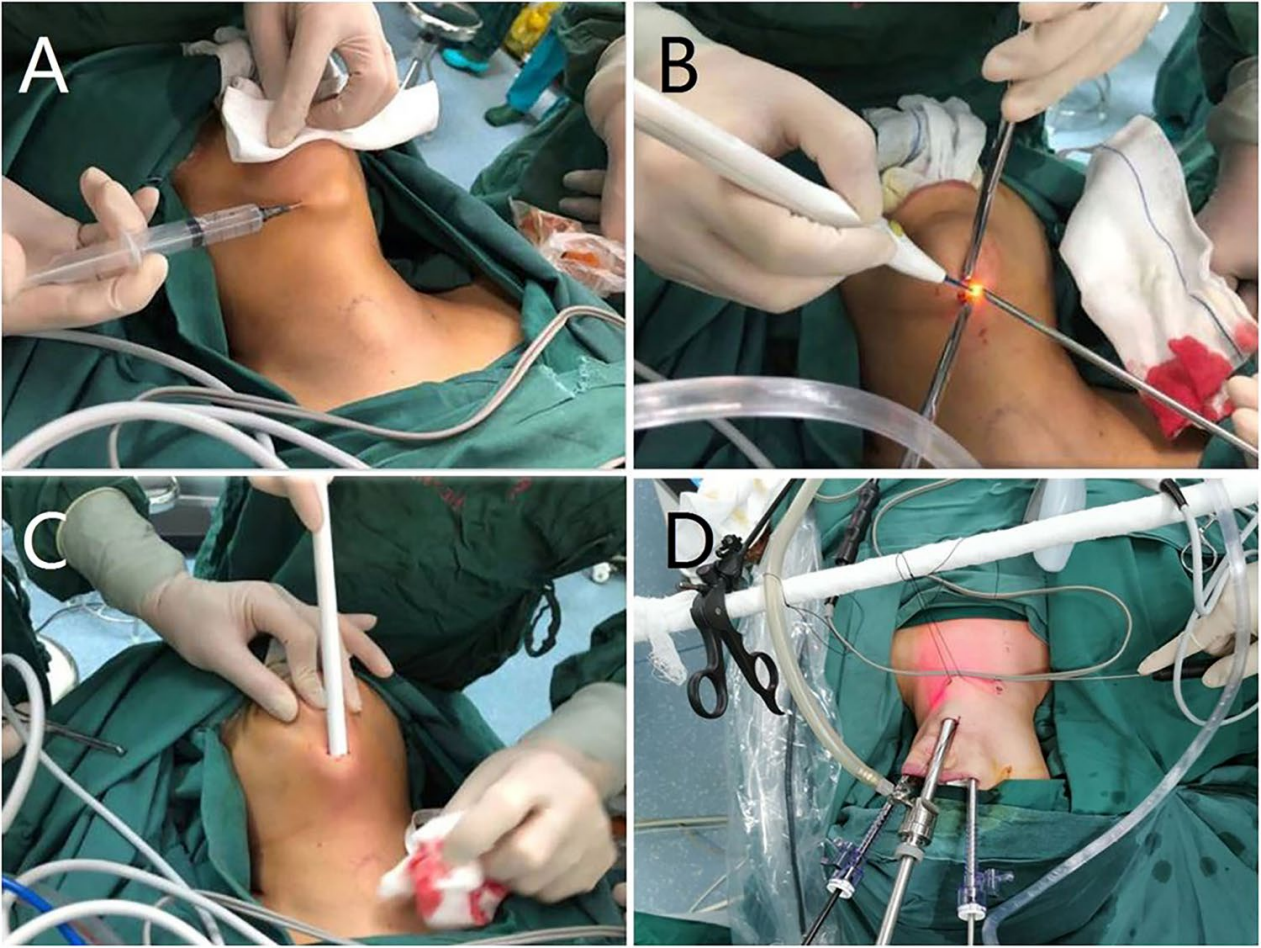
Procedure for establishing the submental incision and placement of trocars. (**A**) Expansion fluid was injected around the designed incision. (**B**) Incising the skin and dissociating to the deep surface of superficial cervical fascia. (**C**) Using blunt separation to establish the space. (**D**) Locations for three trocars; the central trocar was located at the submental incision, and the other two trocars were located at the oral angle

After general anesthesia using a nasal or oral tube, a supine position with shoulder pads raised and head slightly tilted backward was adopted. After disinfecting and spreading the sterile towel, the oral vestibule was disinfected three times, and a saltwater gauze was placed to prevent tongue injury. A 1.5-cm incision was designed at 2 horizontal fingers below the angle of the mandible, and expansion fluid was injected around the designed incision (as shown in Fig. 1A). A needle-like electric knife was used to dissociate to the deep surface of the superficial cervical fascia (as shown in Fig. 1B), and it was further dissociated towards the chest. This level was then bluntly separated with a separator rod to establish a preliminary cavity (as shown in Fig. 1C). Endoscopic trocars were placed, and two 5-mm trocars were placed in the oral vestibule on both sides of the canine teeth, 1 cm from the gingiva. The location and distribution of trocars are shown in Fig. 1D. Two operative trocar incisions were located in the oral vestibule, and endoscopic trocar incision was located under the chin.

The operating space was supported by low-pressure CO_2_ pneumoperitoneum (pressure 4–6 mmHg) combined with external cervical suspension. The separation layer of the cavity was the deep plane of the platysma muscle, and the scope of the cavity was as follows: the lower boundary reached the superior sternal fossa, and the sternocleidomastoid muscle was reached on both sides. After the linea alba was incised, the thyroid surface banded muscle was separated to expose the common carotid artery, and the inferior parathyroid gland was identified and retained in place during separation. Peripheral lymph nodes were visualized, and parathyroid glands were negatively visualized by injecting carbon nanoparticles into the affected thyroid gland. For malignant nodules, the anterior laryngeal lymph nodes were excised first, and then the isthmic region was cut downwards. The upper thyroid pole was isolated, the upper pole blood vessels were closed step by step, and the superior parathyroid gland was identified and retained. Then, the RLN was searched. First, the RLN that enters the larynx was identified; next, the path of the RLN downward was explored. After identifying the neuronal route, Berry’s ligament was cut, and the affected thyroid gland and isthmus were incised. For benign thyroid nodules, the scope of thyroid resection was determined according to the size and distribution of nodules. For single nodules, resection was performed along the edge of nodules, while for multiple nodules, total or subtotal resection was performed.

**Fig. 2:**
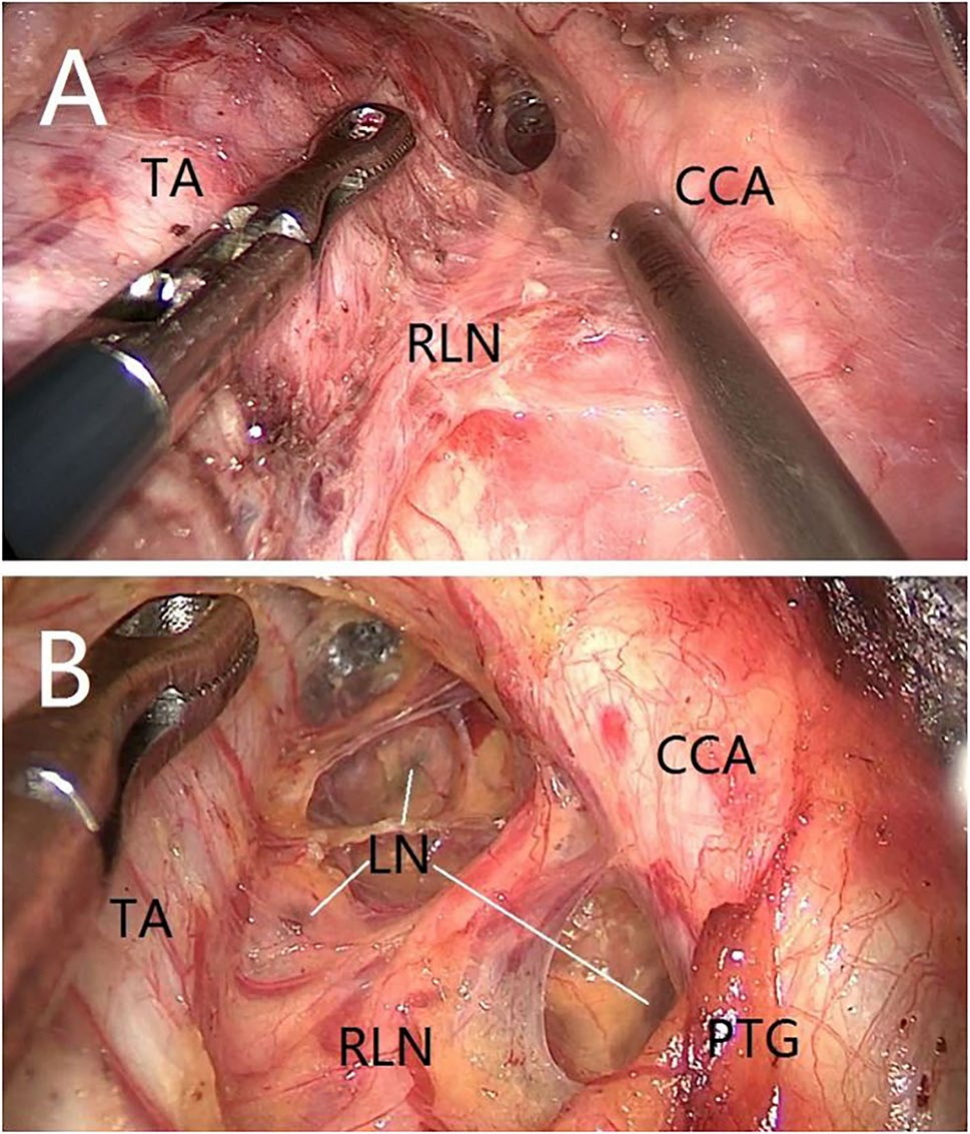
Excellent cranio-caudal endoscopic view of lymph nodes in the central region. TA trachea, CCA common carotid artery, RLN recurrent laryngeal nerve, LN lymph node, PTG parathyroid gland

For patients with thyroid cancer, lymph nodes in the central region were dissected at the same time, ranging from the innominate artery, medial to the opposite margin of the trachea, lateral to the common carotid artery, and deep to the esophagus and prevertebral fascia. Lymph nodes posterior to the RLN were also dissected for right thyroid cancer. As shown in Fig. 2A, the lower boundary of the central lymph node could be clearly seen from a top-down view. Such an excellent field also facilitated the dissection of lymph nodes posterior to the RLN (as shown in Fig. 2B).

**Fig. 3:**
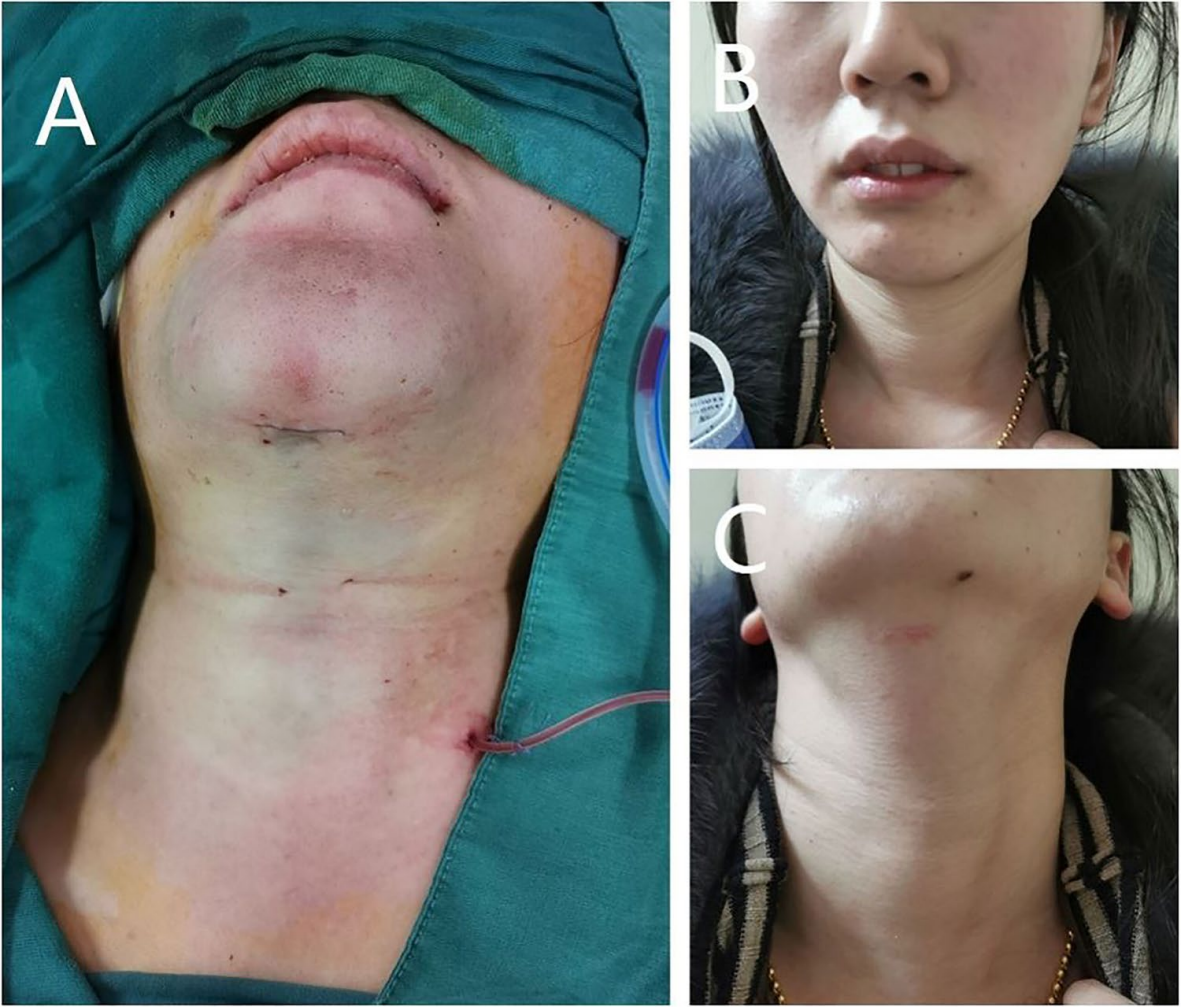
Postoperative cosmetic effect of TOaST. (**A**) The submental incision was sutured after surgery, and a 2-mm drainage tube was placed. (**B**) The patient’s normal posture at 3 months postoperative. (**C**) Submental incision scar 3 months postoperatively

After the specimen was removed from the submental incision, the surgical area was rinsed with sterilized injection water, and the band muscle was sutured with an absorbable thread. Postoperatively, a drainage tube with a diameter of 2 mm was placed, and the oral vestibular and submental incisions were sutured with absorbable suture (as shown in Fig. 3A).

## Results

From December 2020 to December 2021, 54 cases of TOaST were performed in the Department of Thyroid Surgery of the First Affiliated Hospital of Nanchang University. All of the operations were successfully completed, and no patient was transferred to open surgery. All patients recovered well after surgery. The general situation and disease characteristics of all patients are shown in Table 1. There was only one male patient, and all remaining patients were female, with an average age of 36.15 ± 8.78 years (range: 16–54 years). There were 5 cases of American Society of Anesthesiologists (ASA) grade I, 49 cases of ASA grade II, and none of the patients with ASA grade III or above. The mean body mass index (BMI) was 21.67 ± 3.47 (range: 15.82–33.15). There were 27 cases of benign thyroid nodules and 27 cases of differentiated thyroid carcinoma (all papillary carcinoma). The mean maximal diameter of the dominant nodule was 1.89 ± 1.42 cm (range: 0.3–5 cm). In terms of comorbidities, 1 of the patients had hypertension, and his blood pressure was well controlled before surgery. The other patient had associated depression and remained asymptomatic for 1 year before surgery. In addition, 5 patients had mild Hashimoto’s thyroiditis with normal thyroid function.

**Table 1:** General clinical data

	Value
Gender (female/male)	53/1
Age (years)	36.15 ± 8.78
ASA classification (number, rate)	
I	5, 9.26%
II	49, 90.74%
III or above	0
BMI	21.67 ± 3.47
Disease type (number, rate)	
Benign nodules	27, 50%
DTC	27, 50%
Maximal diameter of the dominant nodule (cm)	1.89 ± 1.42
Comorbidity (number, rate)	
Hypertension	1, 1.85%
HT	5, 9.26%
Depression	1, 1.85%

*BMI* body mass index, *DTC* differentiated thyroid carcinoma, *HT* Hashimoto’s thyroiditis

Operation information is shown in Table 2. Among the total 54 patients, 23 patients underwent unilateral subtotal thyroidectomy, 3 patients underwent bilateral subtotal thyroidectomy, 27 patients with unilateral thyroid cancer underwent affected thyroid + isthmus + central lymph node resection, and only 1 patient underwent total thyroidectomy. The mean operative time was 88.06 ± 12.03 min (range: 65–135 min), the mean intraoperative blood loss was 8.61 ± 4.60 ml (range: 5–20 ml), the mean postoperative drainage volume was 49.96 ± 9.88 ml (range: 30–60 ml), the mean drainage time was 36.61 ± 2.65 h (range: 32–50 h), and the mean length of hospital stay was 46.63 ± 3.28 h (range 45–70 h).

**Table 2:** Surgical parameters

	Value
Thyroidectomy range (number, rate)	
Unilateral subtotal thyroidectomy	23, 42.59%
Bilateral subtotal thyroidectomy	3, 5.56%
Affected thyroid + isthmus + central lymph node resection	27, 50%
Total thyroidectomy	1, 1.85%
Operative time (min)	88.06 ± 12.03
Intraoperative blood loss (ml)	8.61 ± 4.60
Postoperative drainage volume (ml)	49.96 ± 9.88
Drainage time (h)	36.61 ± 2.65
Length of hospital stay (h)	46.63 ± 3.28

Surgery-related complications are shown in Table 3. No serious complications occurred in all patients, and 1 patient developed postoperative hoarseness lasting for 1 month, which was diagnosed as postoperative transient RLN injury. Another patient treated with total thyroidectomy presented with postoperative hypocalcemia (hand and foot cramps and facial numbness in the morning), which lasted for 2 months and was diagnosed as postoperative parathyroid dysfunction. There was no superior laryngeal nerve injury and other complications, such as postoperative subcutaneous hematoma, hypercapnia, mental nerve injury, tracheoesophageal injury, infection, or lymphatic leakage.

**Table 3:** Surgery-related complications

	Number, rate
RLN injury	
Transient	1, 1.85%
Permanent	0
Parathyroid dysfunction	
Transient	1, 1.85%
Permanent	0
Superior laryngeal nerve injury	0
Subcutaneous hematoma	0
Hypercapnia	0
Mental nerve injury	0
Tracheoesophageal injury	0
Infection	0
Lymphatic leakage	0

In addition to the surgical efficacy and safety, we also carried out a follow-up of the postoperative cosmetic effect. As shown in Fig. 3B−C, the surgical scar on the patient’s body surface was located under the chin, with a length of about 1.5 cm. When the patient was re-examined 3 months after surgery, the surgical scar could not be seen under the normal posture.

## Discussion

In recent years, the detection rate of thyroid diseases has been increasing annually, and they are mostly found in females and younger people [7]. The number of patients requiring surgery is also increasing. Societal development has led to patients paying increasing attention to the surgical scar, which promotes the rapid development of minimally invasive endoscopic surgery. Minimally invasive endoscopic thyroid-related surgery began in 1996 and was reported by Gagner et al. [8]. To date, there have been a variety of approaches for endoscopic thyroid surgery, including oral [1, 9], axillary [10, 11], and transthoracic mammary [12]. Different approaches have their advantages, and the advantage of the axillary approach lies in less subcutaneous adhesion in the anterior cervical region. The advantages of the transthoracic mammary approach are a large operating space, a short learning curve, and bilateral thyroid management. Furthermore, the advantages of the oral approach lie in the short subcutaneous separation path, no surface scar, in line with the principle of NOTES, and convenience for thorough dissection of central lymph nodes. The unique advantages of the transoral approach make it one of the most popular endoscopic thyroidectomy options.

In 2009, Benhidjeb et al. [1] reported for the first time that transoral endoscopic thyroidectomy was performed by placing a central endoscope 10-mm trocar at the bottom of the mouth and two 5-mm trocars at both corners of the mouth. This approach caused great oral discomfort and postoperative feeding problems. In 2014, Wang [13], a Chinese general surgeon, combined with the characteristics of the Asian flat mandible, set a central 10-mm trocar in the oral vestibule, effectively solving the patients’ oral discomfort and eating problems. In the past 10 years, this method has been rapidly promoted all over the world. This method has become one of the most popular endoscopic thyroidectomy method among surgeons and patients [14]. However, the oral vestibular approach also has some disadvantages, such as the following: (1) during the establishment of the oral vestibular incision, the mental nerve can be easily damaged, and it can cause paresthesia in the mandibular region [15]; (2) the oral vestibular incision changes the type I thyroid surgery into type II thyroid surgery, and many studies have shown that the oral vestibular approach will cause postoperative cervical anterior area infection [16]; (3) the turning point of the mandible will aggravate the difficulty of specimen extraction [17].

To effectively solve these problems, Chen et al. [6] designed an improved operation called TOaST in 2018. The trocar position of central endoscopy was moved out of the oral vestibule and the 5-mm trocar position was kept unchanged on both sides. The specific plan is to move the oral vestibular incision down to the turning point across the mandible and set it at the turning point two horizontal fingers away from the mandible. The incision size was 1.5 cm, and the trocar channel was still the platysma deep surface. After dissociating to the level of the hyoid bone, the subcutaneous space can be established by blunt separation. This improved approach spans the mandible and directly enters the flat anterior cervical area, reducing the intraoperative subcutaneous separation and avoiding the trocar leverage of the prominent mandible, thereby improving the surgical efficiency to a certain extent. This improvement can effectively avoid the mental nerve plexus and the mandibular muscle group (orbicularis oris muscle, mental muscle, and lower labrum muscle) stretching and injury to reduce patients’ mandibular discomfort after surgery. At the same time, after crossing the mandibular turning point, the specimen removal path is flat and wide, thus, reducing the difficulty in removal. The TOaST also maintains a top-down view, which facilitates thorough dissection of the central lymph node. However, the only disadvantage is that there is a 1.5–2-cm scar on the surface of the body, but the good thing is that the incision is visually concealed in the normal posture. In addition, Chen [6] and Insoo [5] reported that the submental incision in this area is the most frequently selected incision location for liposuction, neck lift, or rhytidectomy, and platysmaplasty; thus, incision design also has a cosmetology basis.

Our retrospective study of 54 patients in our center found that the surgical effect and safety of TOaST could be guaranteed, and the surgical scope and lymph node dissection effect of open surgery was achieved. There was only 1 case of transient laryngeal recurrent nerve injury (postoperative hoarseness symptoms disappeared 1 month after surgery) and 1 case of transient parathyroid function injury (the patients underwent total thyroidectomy. On the morning of the second day, there was hand and feet numbness and cramps, and intravenous push calcium gluconate injection improved the symptoms after 2 g. Parathyroid hormone (PTH) and electrolyte tests confirmed hypoparathyroidism; calcium gluconate injection 2 g Bid was given intravenously during the following stay, and calcium carbonate 0.6 g Bid was given after discharge. Two weeks later, PTH and electrolyte levels were significantly improved, and calcium carbonate dosage was gradually reduced. Two months later, the parathyroid function injury had completely recovered). Although the results of this procedure are evident in our center, the results from more centers are needed for the development of this procedure.

## Conclusion

We found that TOaST could not only achieve a good therapeutic effect but also avoid mental nerve injury, reduce the discomfort of the patient’s jaw, obtain a good cosmetic effect, and facilitate the operation of the operator. Thus, it is an endoscopic thyroidectomy technique with a certain clinical value.

## Supplementary Information

Below is the link to the electronic supplementary material.

Supplementary file1 (XLSX 14 KB)
